# Zika virus-like particle (VLP) based vaccine

**DOI:** 10.1371/journal.pntd.0005608

**Published:** 2017-05-08

**Authors:** Hélène Boigard, Alexandra Alimova, George R. Martin, Al Katz, Paul Gottlieb, Jose M. Galarza

**Affiliations:** 1 TechnoVax, Inc., Tarrytown, NY, United States of America; 2 Department of Molecular, Cellular and Biomedical Sciences, City University of New York, School of Medicine, New York City, NY, United States of America; 3 Physics Department, City College of New York, New York City, NY, United States of America; Colorado State University, UNITED STATES

## Abstract

The newly emerged mosquito-borne Zika virus poses a major public challenge due to its ability to cause significant birth defects and neurological disorders. The impact of sexual transmission is unclear but raises further concerns about virus dissemination. No specific treatment or vaccine is currently available, thus the development of a safe and effective vaccine is paramount. Here we describe a novel strategy to assemble Zika virus-like particles (VLPs) by co-expressing the structural (CprME) and non-structural (NS2B/NS3) proteins, and demonstrate their effectiveness as vaccines. VLPs are produced in a suspension culture of mammalian cells and self-assembled into particles closely resembling Zika viruses as shown by electron microscopy studies. We tested various VLP vaccines and compared them to analogous compositions of an inactivated Zika virus (In-ZIKV) used as a reference. VLP immunizations elicited high titers of antibodies, as did the In-ZIKV controls. However, in mice the VLP vaccine stimulated significantly higher virus neutralizing antibody titers than comparable formulations of the In-ZIKV vaccine. The serum neutralizing activity elicited by the VLP vaccine was enhanced using a higher VLP dose and with the addition of an adjuvant, reaching neutralizing titers greater than those detected in the serum of a patient who recovered from a Zika infection in Brazil in 2015. Discrepancies in neutralization levels between the VLP vaccine and the In-ZIKV suggest that chemical inactivation has deleterious effects on neutralizing epitopes within the E protein. This along with the inability of a VLP vaccine to cause infection makes it a preferable candidate for vaccine development.

## Author summary

Zika is an emerging mosquito-borne infection for which vaccines or specific treatments are not available to combat and control its rapid dissemination and deleterious effects. This work describes a novel strategy for the development of a virus-like particle (VLP) based Zika vaccine and shows its effectiveness when tested in a murine model. VLPs are non-infections mimics of the Zika virus, thus chemical inactivation is not required. A VLP vaccine is able to maintain native epitopes structures and to perform better than an inactivated Zika virus control. Our results show feasibility for the rapid development of a safe and potentially highly effective VLP based Zika vaccine for humans.

## Introduction

Zika fever results from an infection with the Zika virus (ZIKV), which is transmitted to humans by the bite of an infected Aedes mosquito (primarily A. aegypti). Zika virus was isolated for the first time from a Rhesus monkey in the Zika Forest in Uganda in 1947 and later from humans in Africa in 1952 [1]. The ZIKV has been transmitted in Africa for many years through a sylvatic cycle between mosquito vectors and nonhuman primates, with occasional human infections [2]. In recent years, however, epidemics of Zika have resulted from cycles of transmission between vectors and humans resulting in the spread of disease beyond the African continent into French Polynesia and other Pacific regions [3–5].

Since 2015, a dramatic spread of ZIKV that began in Brazil has taken place in South America and the Caribbean Islands with the occurrence of sporadic cases in travelers identified in the USA and Europe [6]. Currently autochthonous infections have been reported in the continental US (Florida) [7]. Infection in most cases is asymptomatic or produces a mild illness. However, contracting the virus during pregnancy is associated with birth defects, primarily microcephaly (defective brain development) as well as eye defects and hearing deficits in the infant [8]. Furthermore, an increase in cases of Guillain-Barre syndrome has been observed following ZIKV infection [9]. The seriousness of these disorders imposes a tremendous burden on public health. In addition to vector transmission, ZIKV is transmitted via sexual contact [10, 11], and by body fluid [12–14]. These facts taken together with its often-asymptomatic nature makes disease control even more difficult.

**Fig 1:**
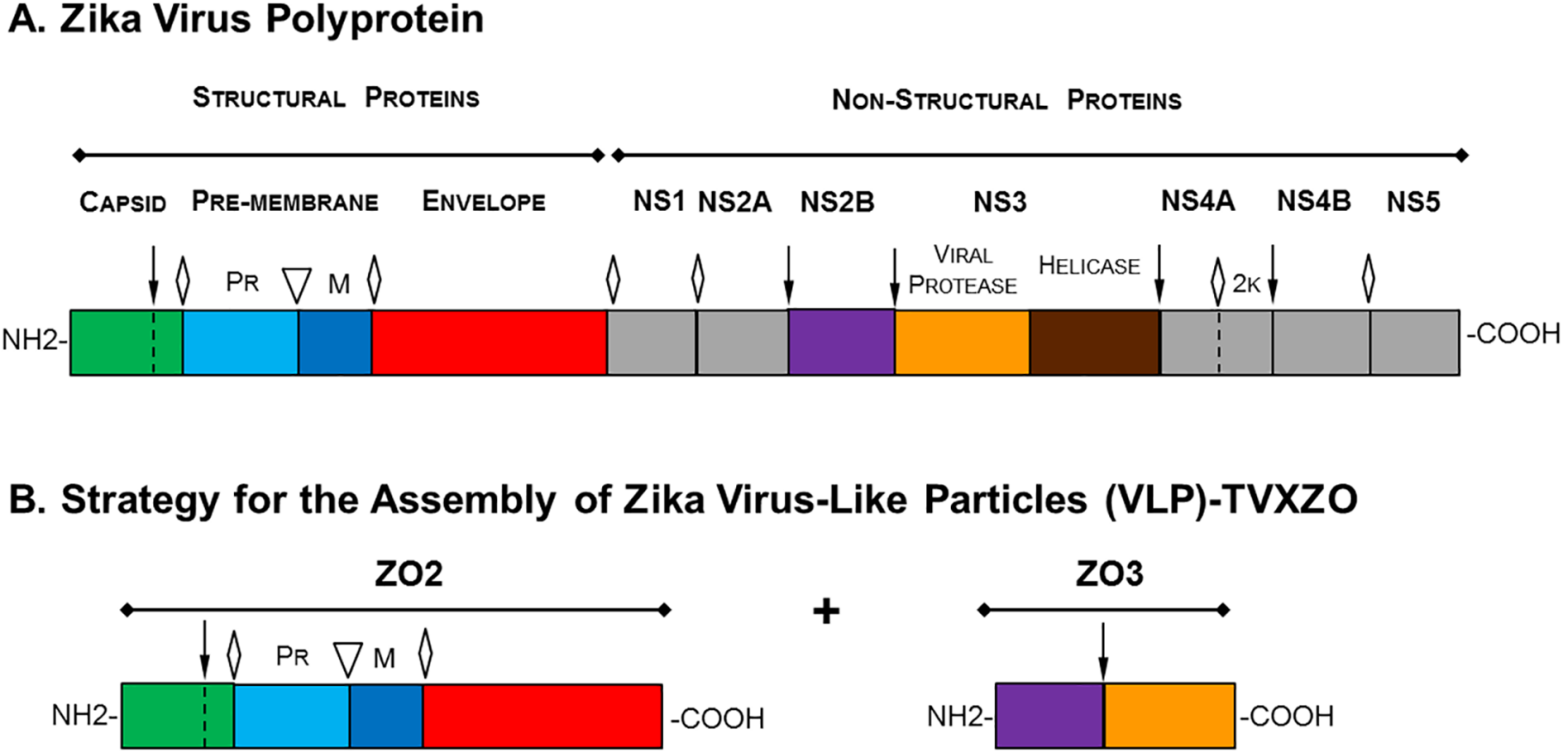
Zika polyprotein processing and strategy for VLP assembly. The schematic depicts the Zika virus genome single open reading frame (ORF) that translates into a polyprotein comprising both structural and non-structural proteins, which arise via several proteolytic cleavages. **(A)** In the early stages, the complex viral protease NS3 with its cofactor NS2B (↓) self-cleaves before cleaving the capsid protein. Host cell signalase is also involved in the maturation of the polyprotein (◊). As a final step, cellular furin cleaves (▽) the pr portion from the M protein to uncover E protein fusion peptide. **(B)** The VLP assembly strategy relies on the co-expression of the structural protein CprME (ZO2) together with the non-structural protein NS2B/NS3Pro (ZO3). The viral NS3 protein is truncated maintaining only its N-terminal protease domain NS3Pro, which is kept as a single transcription unit with its cofactor NS2B. The origin of the sequence encoding these genes and the cloning strategy are described in Material and Methods.

Zika virus is a member of the flavivirus genus within the Flaviviridae family. This family consists of a large group of enveloped viruses, which includes dengue, yellow fever, West Nile, Japanese encephalitis and others that possess a single stranded RNA genome of positive polarity, which serves as mRNA upon the infection of susceptible cells. The ZIKV RNA genome encodes one open reading frame (ORF, ~10,272 nt) and translates into a single polyprotein that similarly to other flaviviruses is co- and post-translationally cleaved by cellular and virus-encoded proteases into three structural proteins (C, prM and E) and into seven non-structural proteins (NS1, NS2A, NS2B, NS3, NS4A, NS4B and NS5.) that enable virus replication (Fig 1A) [15, 16]. Flavivirus replication and morphogenesis occurs in close association with intracellular membranes. Nascent virions are assembled and transported through the secretory pathway and released at the cell surface. Enveloped virions are composed of a cell-derived lipid bilayer encapsulating the C-protein wrapped viral RNA genome and studded with 180 copies of the proteins E and M. During maturation within the secretory pathway the precursor prM protein is cleaved by the host cell furin protease to produce the small M protein and the fragment pr, which is released upon virus egress from the cell. The viral surface displays the E protein as the major antigenic determinant of the virus and mediates receptor binding and fusion during virus entry. Therefore, this protein is a major target for vaccine development [17].

At this time there is no approved vaccine or specific treatment available to control, combat or prevent ZIKV infection. Prophylactic vaccination represents a critical unmet need to address disease spread and its effects globally. Vaccine strategies can rely on inactivated, live attenuated, viral vectors or DNA based compositions, and some of these approaches have been recently tested for Zika vaccine development [18]. However, safety concerns may limit their potential. Here we describe a novel strategy to assemble and produce recombinant Zika virus-like particles (VLPs) and demonstrate their effectiveness when used as a vaccine in an animal model. Development of a safe and efficacious vaccine candidate will provide an effective tool to address this public health emergency.

## Materials and methods

### Genes and plasmids construct

The sequences encoding the structural and non-structural genes of the Zika virus (ZIKV) were chemically synthesized by GeneArt (Life Technology) according to a specifically designed and codon-optimized GenBank sequence: KJ776791.1, derived from Zika virus strain H/PF/2013 genome. This viral genome sequence was the most contemporary available at the time we initiated this work. The synthesized DNA fragments were subcloned into the plasmid vector pcDNA3.4 (Life Technologies) utilizing the XbaI/EcoRV restriction enzyme sites.

The non-structural genes of NS2B and viral protease NS3 were synthesized as a single codon optimized unit and subcloned into vector pcDNA3.4 via NheI/AgeI.

Plasmids were amplified in MAX Efficiency Stbl2 Competent E. Coli Cells (Life Technologies 10268–019) and purified using an EndoFree Plasmid Maxi Kit (Qiagen, MD).

### Virus, cells, and antibodies

Cultures of Vero cells (ATCC CCL-81) were maintained in VP-SFM media (Life Technologies, CA) supplemented with 2 mM L-Glutamine, 2 mM GlutaMAX Supplement (Life Technologies, CA), 1X Non-essential amino acids solution (Life Technologies, CA), 1X ITSE (InVitria) and 500 ng/ml rhEGF (Life Technologies, CA).

#### Zika viruses

ZIKV FSS 13025, a 2013 isolate, was obtained from The University of Texas Medical Branch (UTMB) Arbovirus Reference Collection (# TVP 22623 UTMB, Galveston, TX) and amplified in Vero cells following a low multiplicity of virus infection (MOI: 0.01).

ZIKV MR-766 was obtained from the American Type Culture Collection (ATCC) (#NR-50065, Manassas VA) and also amplified in Vero cells at ~MOI: 0.01 and harvested 96 h post infection. The inactivated Zika virus control (In-ZIKV) was prepared using the MR-766 strain. Purified virus (see below) was inactivated with formalin at the final concentration of 0.01% and incubated at 37°C for 24 h [19]. Work with Zika virus was conducted under biosafety level 2 (BSL2) containment and procedures.

Expi293 (Life Technologies, CA) cells were expanded in Expi293 medium (Life Technologies,) and transfected using Expifectamine following the manufacturer instructions (Life Technologies, CA). Monoclonal antibody 4G2 is an in-house produced and protein G purified preparation from hybridomas D1-4G2-4-15 culture supernatant (ATCC HB-112). Monoclonal antibodies ZV-48, ZV-64 and ZV-67 were kindly provided by Dr. M. S. Diamond [20]. Rabbit polyclonal antibodies, anti-M (GTX133305), anti-E (GTX133314), anti-NS2B (GTX133308) and anti-NS3 (GTX133309) were purchased from GeneTex, CA.

Horseradish peroxidase conjugated secondary antibodies anti-mouse and anti-rabbit were purchased from Pierce Thermo Fisher, MA (#31430 and# 31460 respectively). Alkaline-phosphatase conjugated secondary goat anti-mouse antibody was purchased from Pierce Thermo Fisher, MA (#31330).

The human serum was obtained from Kerafast (#EVU302) Boston, MA. The laboratory of James E. Crowe Jr., MD, Vanderbilt University, collected this serum from an otherwise healthy donor who contracted laboratory confirmed Zika virus infection in July 2015 following natural exposure to mosquitoes in Brazil.

### VLP production and purification

Expi293 cells in suspension culture were transfected with a 1:2 ratio of pcDNA3.4-NS2b/NS3 (ZO3) and pcDNA3.4-CprME (ZO2) at 37°C in a 5% CO_2_ environment and agitated at 150 rpm. Transfected cells were harvested 72 h post-transfection and clarified via two successive centrifugations. The first clarification was performed at 400g for 10 min at 4°C followed by a second clarification at 10,000g for 10 min at 4°C. The remaining proteins in the supernatant were precipitated using 8% (w/v) of polyethylene glycol 8000 and incubated overnight at 4°C. A protein pellet was collected after centrifugation at 14,000g for 30 min at 4°C and loaded onto a 20% w/v sucrose cushion in TNE buffer (10 mM Tris-HCl, pH 8.0, 120 mM NaCl and 1 mM EDTA) and spun by ultracentrifugation at 150,000g for 2 h at 4°C. The pellet was resuspended in TNE buffer. Viruses and VLP were then purified by ultracentrifugation through a linear potassium tartrate 10–35% (w/v) / glycerol 7–30% (v/v) density gradient at 180,000g for 4 h at 4°C. Fractions were collected and analyzed by dot blot using 4G2 monoclonal mouse antibody. The 4G2 MAb reacts with dengue, Zika and other flavivirus [21] and has been shown to recognize a conformational epitope within domain II of the dengue E protein [17] and presumably recognizes a similar epitope in the Zika virus. Selected fractions were further purified and concentrated using Amicon Ultra Centrifugal Filter Unit (Millipore, MA).

### Dot blot, Western blot and Coomassie blue stain

The cell protein content was analyzed after clarification of transfected Expi293 cells. The cell pellets were lysed with RIPA buffer (Pierce Thermo Fisher, MA) according to the vendor protocol. For Western blotting, cell lysates and concentrated culture supernatants were loaded onto a 4–12% Bis-Tris SDS-polyacrylamide gel (Life Technologies, CA). After electrophoretic separation, proteins were electro-transferred from the gel onto a 0.45 μm nitrocellulose membrane (Life Technologies LC2001). For dot blot analysis, 3 μl of sample was applied to a 0.45 μm nitrocellulose membrane and allowed to dry for 5 min. The nitrocellulose membranes were then blocked with 5% non-fat milk in TBST (10 mM Tris-HCl, pH 7.4, 130 mM NaCl, 2.7 mM KCl and 0.1% Tween-20) for 1 h at room temperature followed by overnight incubation at room temperature in primary antibody diluted with blocking buffer. Membranes were washed 3 times with 1X TBST and then incubated for 2 h with secondary antibody diluted in blocking solution. Finally, membranes were washed 3 times with 1X TBST and developed with ECL system (Life Technologies, CA).

The total protein concentration was determined using the Bradford method. The E protein content in the purified VLPs and inactivated ZIKV (In-ZIKV) was determined using densitometry analysis of Coomassie blue stained SDS-PAGE gels. Different concentrations of a BSA standard were loaded onto the same gel. The stained gel image was acquired with a FluorChem M imager instrument (Protein Simple, CA). The optical density of the bands was analyzed using Alphaview software. The BSA concentrations were used to establish standard curve and parameters of the linear regression curve were used to determine E protein concentration of each VLP vaccine and In-ZIKV. Dot blot were performed with purified and quantified VLP or ZIKV and the amount of sample applied is specified in the corresponding figure legend.

### Negative staining and immuno-gold labeling TEM

VLP or Zika virus samples were prepared for TEM examination as follow: Samples for immunogold labeling TEM (2.5 μl) were loaded onto CF200-CU carbon film 200 mesh copper grid (product of Electron Microscopy Science) and incubated for 5 min at room temperature, then washed with PBS, blocked in 1% BSA in PBS for 5 min and incubated on the surface of a primary antibody drop for 30 min at room temperature. We use two antibodies, the mouse MAb 4G2 diluted 1:500 in PBS and a polyclonal human serum from a Zika virus infected patient diluted 1:20 dilution in PBS. The grids were then washed 6 times with PBS and incubated on the surface of a drop of secondary antibody conjugated with gold beads for 30 min. A goat anti-mouse antibody conjugated with 10 nm gold beads was used with the mouse MAb 4G2 and a goat anti-human antibody conjugated with 6 nm gold beads was used with the human serum. Grids were finally washed 6 times with PBS, and then fixed for 15 min with 4% paraformaldehyde in PBS, washed with PBS and subsequently washed twice with 0.2M Sodium cacodylate buffer and finally stained with 1% uranyl acetate solution.

### Mouse immunogenicity and efficacy study

The aim of the study was to determine the immunogenicity and efficacy of a VLP-based Zika vaccine and its capacity to elicit a strong neutralizing serum protective immune response. Experimental groups comprised eight mice (n = 8) in order to study pairs of subjects while being able to reject a null hypothesis with statistical power of >99% and a standard deviation of 19. The alpha (α) error probability associated with this test is 0.01.

Nine groups of 6 to 8-week old female BALB/c mice (n = 8) were inoculated twice (day 0 and day 24) via the intramuscular (IM) route with either VLP vaccine, formalin inactivated ZIKV MR-766 (In-ZIKV) control or PBS plus adjuvant negative control (Neg. Ctr.). Mice received doses of either 1 μg or 4 μg of total E protein content formulated alone or admixed in a 1:1 volume ratio with a squalene-based oil-in-water nano-emulsion AddaVax (InvivoGen, CA). Serum samples were collected from all animals at day 42.

### Evaluation of serum antibody levels by ELISA

Serum IgG titers against Zika viruses were determined by ELISA. Briefly, assays were performed in 96-well plates coated with 50 μl/well of purified and inactivated Zika virus MR-766 or FSS-13025 (2 μg/ml total protein concentration) at 4°C overnight. Subsequently, plates were washed 3 times with PBS-T (0.05% Tween-20 in phosphate buffered saline) and then blocked with 100 μl of blocking solution (5% non-fat milk in PBS-T) for 1 h at room temperature (RT). Vaccine and control sera were diluted in fourfold series in blocking buffer and applied to each well in triplicate and incubated for 2 h at RT. After 6 washes with PBS-T, plates were incubated for 2 h at RT with 50 μl of HRP-conjugated goat anti-mouse IgG (H+L) secondary antibody diluted (1:5000) in blocking buffer). Subsequently, plates were washed 6 times with PBS-T and developed by adding 50 μl per well of Ultra TMB solution (Thermo Scientific, MA) for 20 min of incubation and stopped with 100 μl of stop solution (2 M HCL solution). ELISA end-point titer for each group was calculated as the reciprocal of the highest dilution with OD value 2σ above the mean of the negative control wells.

### Plaque reduction neutralization test (PRNT)

The plaque reduction neutralization test was carried out in Vero cells using sera from immunized mice and either MR-766, or FSS-13025 Zika viruses following the method described for the evaluation of flavivirus vaccine efficacy [22, 23]. Briefly, ZIKV was amplified and titrated in Vero cells using the same plaque visualization procedure described below. Vero cells were seeded in 24-well plates at a density of 6x10^4^ cells per well 24 h prior to the initiation of the test. Control and test sera were heat inactivated at 56°C for 30 min. Starting at a 1:20 dilution, the sera were two-fold serially diluted with cell culture media supplemented with penicillin and streptomycin. Equal volumes of diluted virus to form ~60 plaques per well were added to each serum dilution. The sera-virus mixtures were incubated for 1 h at 37°C in a 5% CO_2_ environment. Afterwards, each dilution was applied in duplicated to wells of 85% confluent monolayer of Vero cells and incubated for 90 min at 37°C in a 5% CO_2_ incubator. Thereafter, the inocula were removed and a 1 ml overlay of 1.8% carboxymethyl cellulose (CMC) in culture medium was added to each well. Plates were then incubated for 3 days at which time the CMC overlay was removed, cells washed with PBST (phosphate buffer saline plus 0.05% Tween 20) and fixed with cold 80% acetone for 10 min at -20°C. Subsequently, plates were washed with PBST and then incubated with blocking buffer (2.5% non-fat milk in 0.5% of Triton X-100 PBS solution) for 1 h in a 37°C incubator. After one wash, the primary antibody (MAb 4G2) diluted 1:500 in blocking buffer was applied for 2 h at RT, followed by two PBST washes and incubation for 1 h at RT with a goat anti-mouse AP conjugated secondary antibody diluted (1:2000) in blocking buffer. Finally, plates were washed twice with PBST and once with alkaline phosphate buffer (APB: 100 mM Tris-HCl pH9.0, 150 mM NaCl, 1 mM MgCl_2_). Viral plaques were detected by adding the alkaline phosphate (AP) substrate nitro blue tetrazolium chloride (NTB) and 5-brome-4-chlore-3-indolyl phosphate (BCIP) prepared and used as follows: Combine 33 μl of NTB (50 mg/ml in 70% dimethylformamide) with 5 ml of APB mix well and then add 16.5 μl of BCIP (50 mg/ml in 100% dimethylformamide) and the mixture should be used within 1 h of preparation. Plaques were counted and PRNT50s were determined using the PROBIT method [24]. The neutralization power calculated is expressed as the reciprocal of the highest serum dilution that neutralizes 50% of the virus and the histograms represent the average neutralization 50% per group and their standard deviations.

### Antibody-dependent enhancement (ADE) assay of DENV-2 infection

Suspension cultures of U937 cells grown in RPMI supplemented with 10% of FBS were used in the ADE assay, which was performed according to Diamond et. at. [25]. Prior to the initiation of the experiment (72h), the U397 cells were differentiated toward the granulocyte or macrophage lineage by the addition of dimethyl sulfoxide (DMSO; 1.25%) [25]. DENV-2 virus (50μl) was mixed with diluted serum samples (four for each vaccine group) and controls (virus alone or 4G2 MAb-200ng) and then added to 2.5x10^5^ cells resulting in a multiplicity of infection of 3 (MOI: 3). The resulting mixture of cells, virus and serum was incubated for 2 h at 37°C. Subsequently, cells were washed four times to remove free virus, suspended in culture medium and then incubated for 96 h at 37°C. Thereafter, virus titers in the culture supernatants were determined by plaque assay as described above in the PRNT assay.

### Statistics

All statistical calculations were carried out using GraphPad Prism 4 software. Tests between two groups used two-tailed Student’s t-test. The P-value was calculated for <0.05 level of significance.

### Ethical statement

Mouse studies were carried out in the Department of Comparative Medicine, Animal Facility of the New York Medical College, Valhalla, NY. All animals were cared for in compliance with the *Guide for the Care and Use of Laboratory Animals* and all experiments were approved by the New York Medical College’s IACUC. Mice were housed in an AAALAC-accredited facility.

## Results

### Strategy for the assembly of Zika virus-like particles (VLPs)

**Fig 2:**
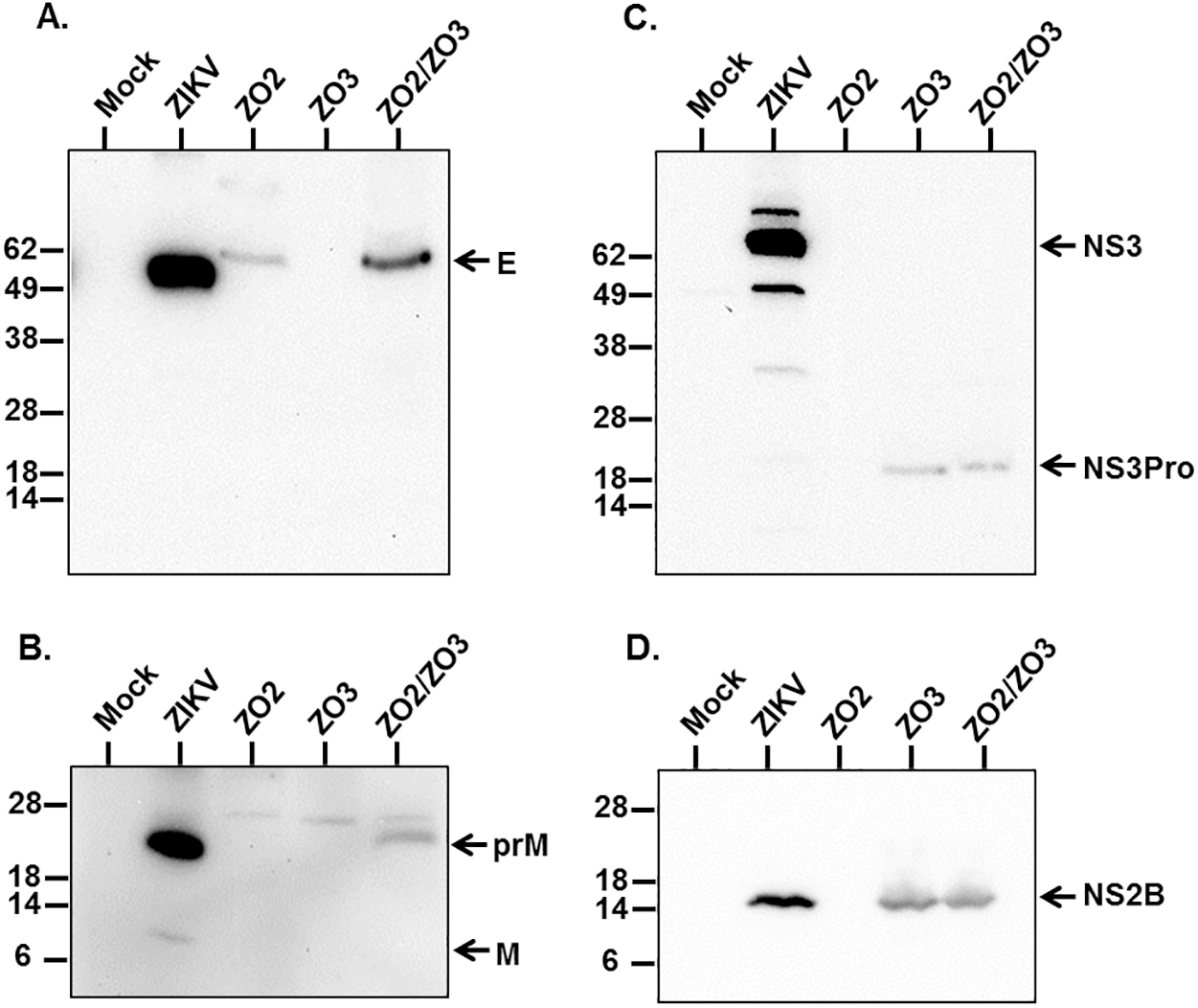
Analysis of protein expression and processing in lysates of transfected cells by Western blot. Cell lysates (30μg total protein) of Expi-HEK293 transfected with plasmids ZO2 (CprME), or ZO3 (NS2B/NS3Pro) or both (ZO2/ZO3) were probed with anti-Zika protein specific polyclonal antibodies (See Material and Methods). Zika virus (ZIKV) infected and uninfected (mock) Vero cell were controls. **(A)** Anti-E specific antibody detected E protein expression and cleavage in ZO2/ZO3 and ZO2 (lesser amount) cell lysates but not in that of ZO3. E was also detected in ZIKV infected cells but not in the mock control. **(B)** The prM protein (~32KDa) was detected in ZO2/ZO3 transfected cells as well as in ZIKV infected cells, which also showed a small amount of M (~ 8KDa). **(C)** NS3Pro (~19KDa) was detected in ZO3 and ZO2/ZO3 transfected cells whereas the full-length protease/helicase NS3 (~69KDa) was detected in ZIKV infected cells. Neither was detected in ZO2 transfected cells or the mock control. **(D)** NS2B (~14KDa) was detected in ZO2 and ZO2/ZO3 transfected cells and ZIKV infected cells whereas ZO2 and mock transfected cells were negative.

Early studies on flavivirus replication have demonstrated that together with complete infectious virions, particles are also produced that lack the viral RNA genome, which have been termed small, non infectious subviral particles (SVPs) or virus-like particles (VLPs) [26]. Assembly of these non-infectious particles using recombinant methods has been utilized to study protein function, morphogenesis and structure [27–29] and to generate flaviviruses vaccines, which have proven to be immunogenic [30]. We have found a new and effective strategy to assemble ZIKA VLPs and utilize them for vaccine development. Formation of VLP is accomplished by the co-expression of the Zika virus structural proteins CprME together with a truncated form of the protease NS3Pro linked to its cofactor NS2B constituting the viral NS2B/NS3Pro protease complex (Fig 1B). Transfection of suspension cultures of Expi-293 cells with plasmids expressing CprME and NS2B/NS3Pro produces the structural polypeptide CprME, which undergoes proteolytic cleavages mediated by cellular and the co-expressed NS2B/NS3Pro proteases (Fig 1B). Expression of NS2B/NS3Pro and processing of the polyprotein CprME was evaluated by Western blot analysis using lysates of cells transfected with a single plasmid expressing CprME (ZO2) or a single plasmid expressing NS2B/NS3Pro (ZO3) or a combination of both plasmids. This analysis showed that the protease NS2B/NS3Pro was expressed and self-cleaved rendering NS2B (~14KDa) (Fig 2D) and NS3Pro (~19KDa) (Fig 2C) when cells were transfected with the plasmid ZO3 alone (expressing NS2B/NS3Pro) or together with ZO2 (expressing CprME) but not in cells only transfected with the plasmid ZO2 (CprME) (Fig 2C and 2D). Analysis of a ZIKV infected Vero cell lysate revealed the expression of the full-length NS3 protein (containing both the protease and the helicase domains), which was absent from the lysate of uninfected (mock) Vero cells (Fig 2C). Similarly, Western blot studies demonstrated that the polyprotein CprME was expressed and processed yielding the expected proteins E and prM, as well as intermediate cleavage products including prM and CprM (Fig 2A and 2B). Release of prM and C from ZO2 alone seems to occur at a lower frequency in the absence of the virus protease complex NS2B/NS3Pro. Therefore to detect M and pr in ZO2 alone requires higher amount of loaded material than the one used (Fig 2B). We were unable to detect the C protein in neither the ZIKV infected cell lysate nor the VLP transfected lysates using the currently available anti-C antibody. However, the detection of E and prM suggests that C is cleaved as well. This analysis demonstrates protein expression as well as the activity of NS2B/NS3Pro, which is able to self-cleave uncoupling NS2B and NS3Pro before subsequently cutting of C from the polyprotein (Figs 1 and 2A–2D). Further cleavage processing steps mediated by cellular signalase proteases separate E from M and C from pr. The released E protein shows slightly greater size than the E of Zika MR-766 virus infected Vero cells (Fig 2A), due to the lack in this ZIKV strain of four amino acids in the E protein (153–156) including the glycosylation site asparagine (N-154), which are present in the E protein of ZIKV H/PF/2013 used for VLP production as well as other ZIKV strains such as the more contemporary strain FSS-13025 used as control [31]. At the time of immunization, only the viral strain MR-766 was available.

### Production and characterization of Zika virus-like particles (VLPs)

**Fig 3:**
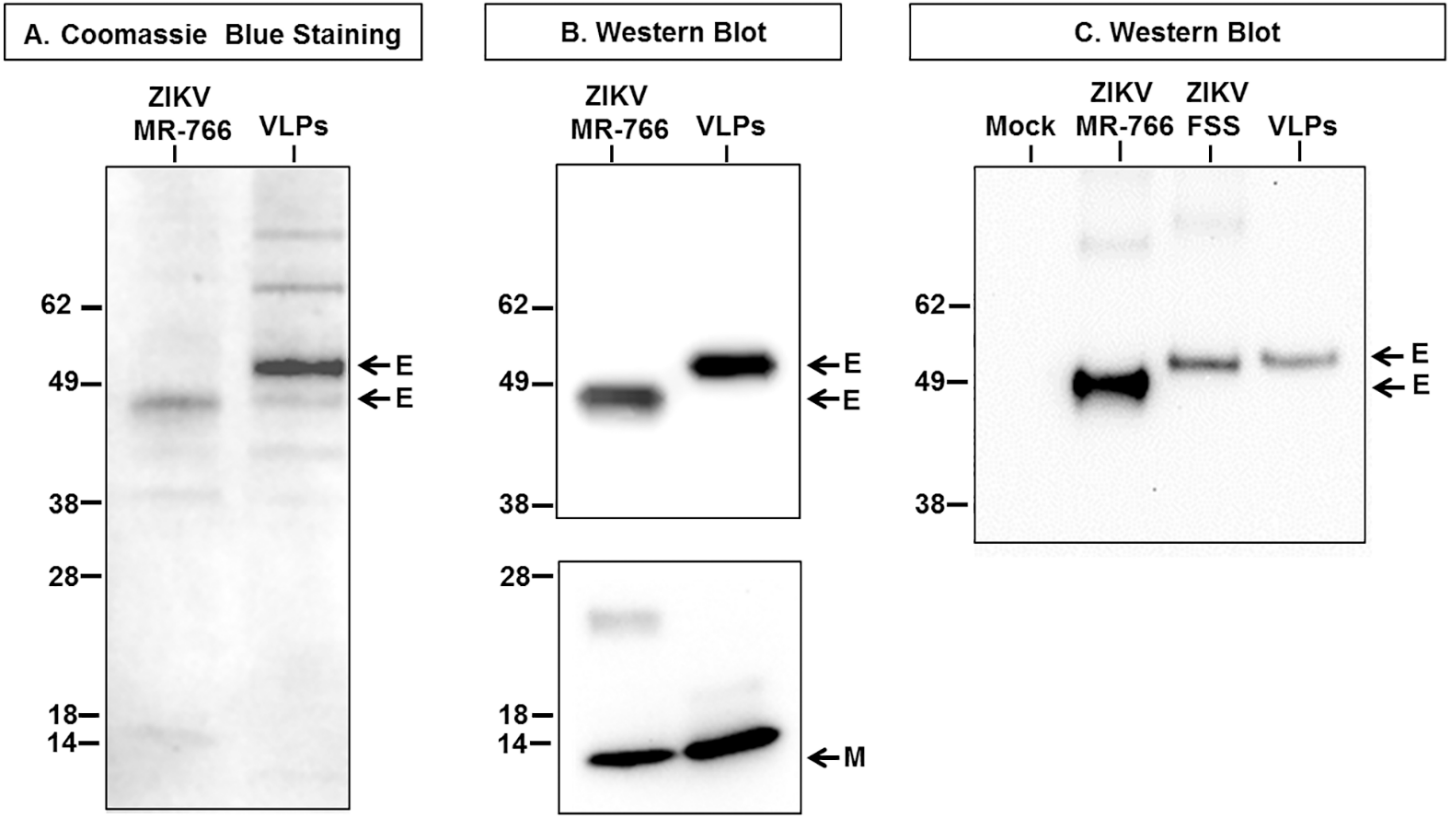
Analysis of purified VLPs and ZIKV by Coomassie blue stain and Western blot. A total of 1.8 μg of purified VLPs or ZIKV were resolved by SDS-PAGE and protein content and identity analyzed by **(A)** Coomassie stain and **(B)** Western blot using Zika protein specific antibodies, anti-E upper panel and anti-M lower panel **(C)** Comparison of the size of the E protein of ZIKV MR-766; FSS-13025 and VLPs by Western blot.

The recombinant production of Zika VLPs was carried out in suspension cultures of Expi-HEK293 cells following co-transfection of the plasmids ZO2 (CprME) and ZO3 (NS2B/NS3 Pro). The VLPs were harvested from the culture supernatant and purified as described in Material and Methods. The Zika virus grown in Vero cells was subjected to an analogous purification scheme as the one described for the VLPs. The purity and identity of the protein component of the purified VLP and Zika virus were assessed by Coomassie blue staining and Western blot analysis. As shown in Fig 3A, the E protein was readily detected as the predominant component of both the ZIKV and the VLP preparations. The identity of the E protein was verified by Western blot analysis and exactly correlated with the E protein visualized in the stained gel (Fig 3B). The migration difference of the E proteins detected in the cell lysates was also seen between the E protein of the purified ZIKV-MR-766 and VLPs (Fig 3A and 3B). This disparity, as noted above, is due to the lack of four amino acids including the glycosylation site N-154 in ZIKV-MR-766. These residues are present in ZIKV-FSS-13025 and H/PF/2013 and sequences of this strain were used to produce the VLPs. Examination by Western blot of the migration patterns of the E protein from ZIKV-MR766, FSS-13025 and VLPs (genes derived from H/PF/2013) showed that the MR-766 E protein migrated slightly faster than the E protein from ZIKV FSS-13025 and VLPs, which exhibit similar size (Fig 3C). Coomassie staining also showed minor unidentified proteins in the VLP preparation that were also present in the ZIKV albeit in lesser amounts (Fig 3A). The M protein, a major structural component of the Zika virus, was clearly identified by Western blot analysis in both the ZIKV and VLP preparations but it was not clearly seen in the stained gel, possibly due to its small size and low concentration, which precluded retention of sufficient amounts of bound dye for clear detection (Fig 3A). Both, E and M the major structural proteins were clearly identified by Western blot (Fig 3B).

**Fig 4:**
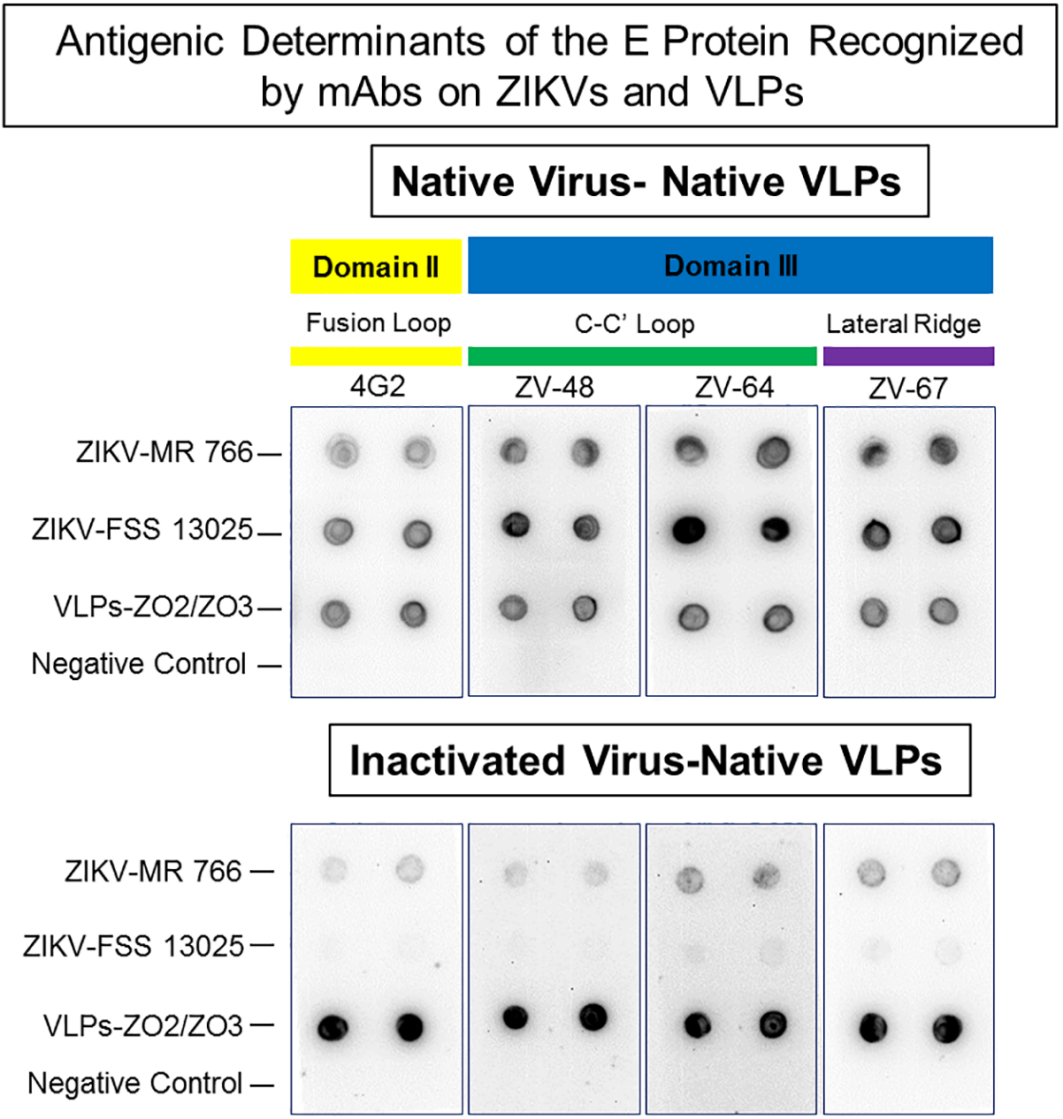
Examination of reactivity of native VLPs and native and inactivated ZIKV with monoclonal antibodies recognizing structural epitopes. VLPs were produced and purified as described in material and methods. ZIKV MR-766 and FSS-13025 were grown in Vero cells, PEG-8000 precipitated and concentrated by centrifugation through a 20% sucrose cushion. Half of the virus sample was inactivated with formalin and then treated and untreated (native) virus samples were further purified via density gradient centrifugation. Dot blot analysis of purified VLPs and ZIKVs (0.2μg/μl, 3μl per dot of E protein content) were probed the MAbs, 4G2, ZV-48, ZV-64 and ZV-67, which recognize conformational epitopes in distinct domains of the E protein, showed that VLPs and native ZIKVs reacted with the MAbs, whereas the reactivity of formalin inactivated ZIKVs virus was greatly reduced as compared to native VLPs. This outcome suggests that formalin has a deleterious effect on the ZIKV conformational epitope recognized by these MAbs.

Furthermore, to corroborate whether conformational neutralizing epitopes of the E protein were exhibited on the VLPs, we probed by dot blot purified VLPs and ZIKVs with the domain specific neutralizing MAbs, ZV-48, ZV-64 and ZV-67 [20] in addition to the mouse MAb 4G2. This examination showed that the VLPs were indeed recognized by these MAbs indicating that these neutralizing epitopes were properly displayed in the VLPs as they also were in the ZIKV MR-766 and FSS-13025 (Fig 4). We have also observed that the reactivity of Zika viruses with these MAbs is mostly abrogated after the viruses are inactivated with formaldehyde (Fig 4). The VLP sample remained untreated.

### Negative staining and immunogold labeling electron microscopy

**Fig 5:**
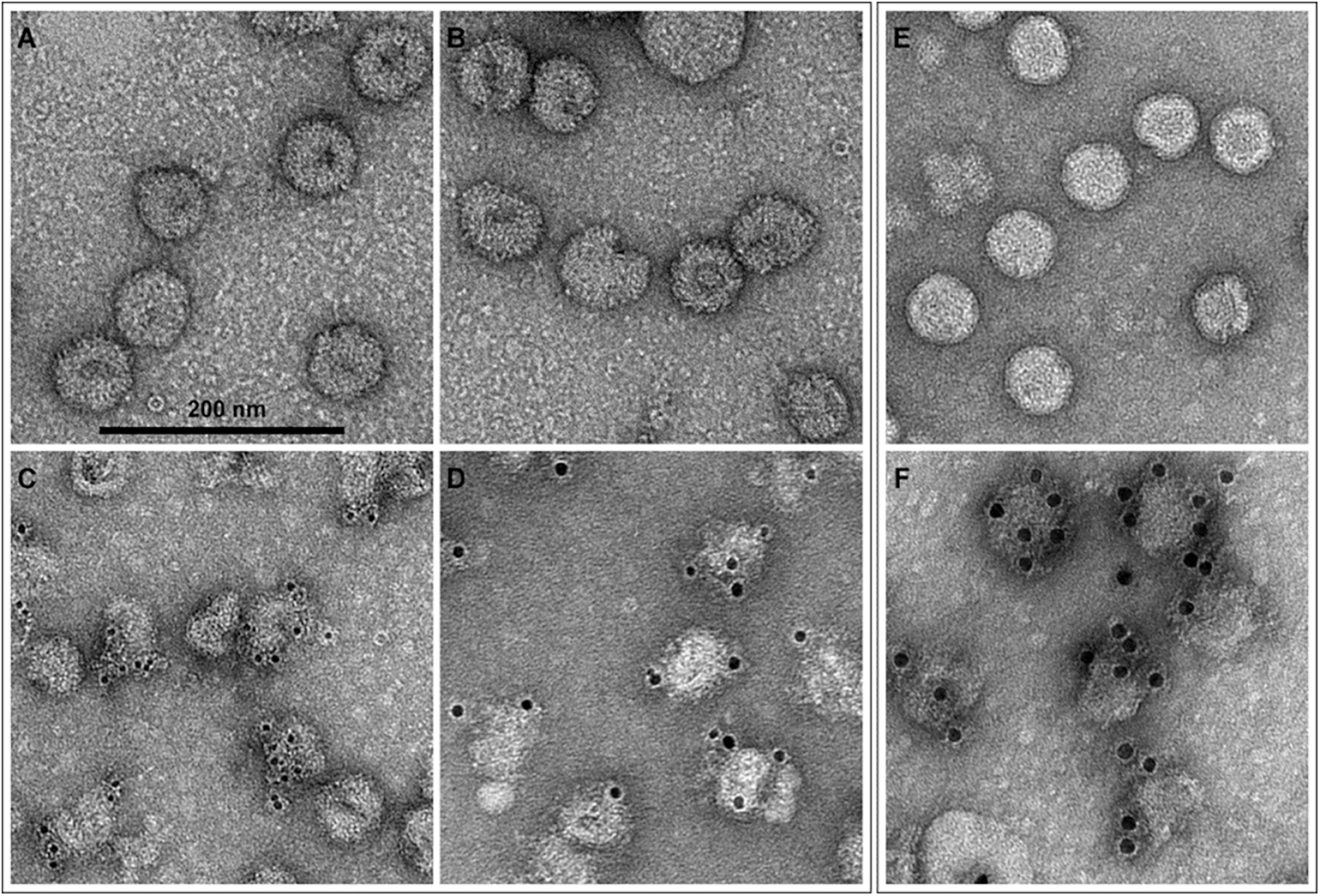
Electron micrographs of negative staining and immunogold labeling of VLPs and ZIKV. Purified VLPs and ZIKV were examined by transmission electron microscopy (TEM). Panels A and B show uranyl acetate negatively stained VLPs which exhibit particle sizes from 50nm to 65nm in diameter (mean 60nm) and a structure that resembles the morphology and surface appearance of wild type Zika virus which is shown in Panel E. Immunogold labeling with two antibodies, mouse anti-E protein MAb 4G2 and a human serum from a Zika patient served as primary and counterstained with anti-mouse or anti-human secondary antibody conjugated with gold beads of 6 nm and 10nm in diameter, respectively. Both antibodies, 4G2 panel C and human serum panel D, bind to the particle surfaces as revealed by the presence of gold beads. Panel F shows wild-type ZIKV probed with the 4G2 antibody, which also binds the virus surface as revealed by the detection of gold beads; in this case goat anti-mouse secondary antibody conjugated with 10 nm gold bead was applied. These studies demonstrate that a specific anti-E MAb and an anti-Zika polyclonal antibody reacts with the VLP surfaces indicating that the major Zika surface antigen, the E glycoprotein, is indeed displayed on the VLP surfaces.

To further characterize the structure and surface composition of the VLPs, we examined purified material by transmission electron microscopy (TEM). Negative staining studies revealed that the VLPs are fairly homogeneous spherical structures with an average diameter of 60 nm. The VLP surfaces appeared smooth without noticeable projection or rough features. Comparative analysis with purified ZIKV showed that both VLP and virus particles are similar in size, morphology and surface appearance (Fig 5A, 5B and 5E). This is in agreement with recent reports of the Zika virus structure [31, 32]. To better define the surface composition of the VLPs, we probed the recombinant particles by immunogold labeling with two distinct antibodies, 4G2, a MAb that recognizes a conformational loop in domain II of the E protein shared by some members of the flavivirus family including Zika [21], and a polyclonal human serum from a Zika patient. Subsequently these preparations were examined by negative staining TEM. These studies demonstrated the reactivity of the VLPs with both antibodies (Fig 5C and 5D) indicating not only that the E protein is displayed on the surface of the particles but that it also maintains its native conformation based on reactivity with the MAb 4G2 (Fig 5C). Examination of purified ZIKV also showed equivalent reactivity with the 4G2 antibody (Fig 5F) confirming that both the VLP and live ZIKV exhibit on their surfaces the conformational site recognized by 4G2 (Fig 5C and 5F). We further probed VLPs with the serum from a Zika patient and found that it also binds to the particles’ surface providing addition evidence that the ZIKV major surface glycoprotein E is indeed present on the VLP surface (Fig 5D).

### VLP based Zika vaccine evaluation

We sought to assemble Zika VLPs with the main objective of developing a safe and effective vaccine to stem the rapidly spreading Zika epidemic. To establish immunogenicity and in-vitro efficacy of a VLP-based Zika vaccine, we designed a mouse study to assess the performance of a VLP vaccine and to compare the outcome to an equivalent formulation and dose of an inactivated Zika virus (In-ZIKV) control. Given the urgency of advancing a Zika vaccine and the lack, at the onset of the study, of well-established Zika animal models, we selected a mouse model to carry out this vaccine evaluation. Two groups of 6 to 8- week old female Balb/c mice (n = 8 each) were immunized via the intramuscular route (IM) twice, three weeks apart, with two difference doses, 1 μg and 4 μg of total E protein of VLP vaccine formulated with or without adjuvant (AddaVax, InVivoGen) a squalene-oil-in water nano-emulsion that stimulates a balanced immune response and has a formulation similar to MF59, a licensed adjuvant in flu vaccines in Europe [33, 34]. Similar groups of mice (n = 8 each) were immunized via the same route and schedule with a formalin inactivated Zika virus (In-ZIKV) control at the doses of either 1 μg or 4 μg of total E protein content also formulated with or without adjuvant. A negative control (Neg. Ctr.) group (n = 8) received phosphate buffered saline (PBS) plus adjuvant following the same immunization regimen as used for the vaccine groups. Three weeks after the booster immunization, animals in all groups were terminally bled and sera samples prepared for immunogenicity and efficacy evaluation.

### Evaluation of immunogenicity by ELISA

**Fig 6:**
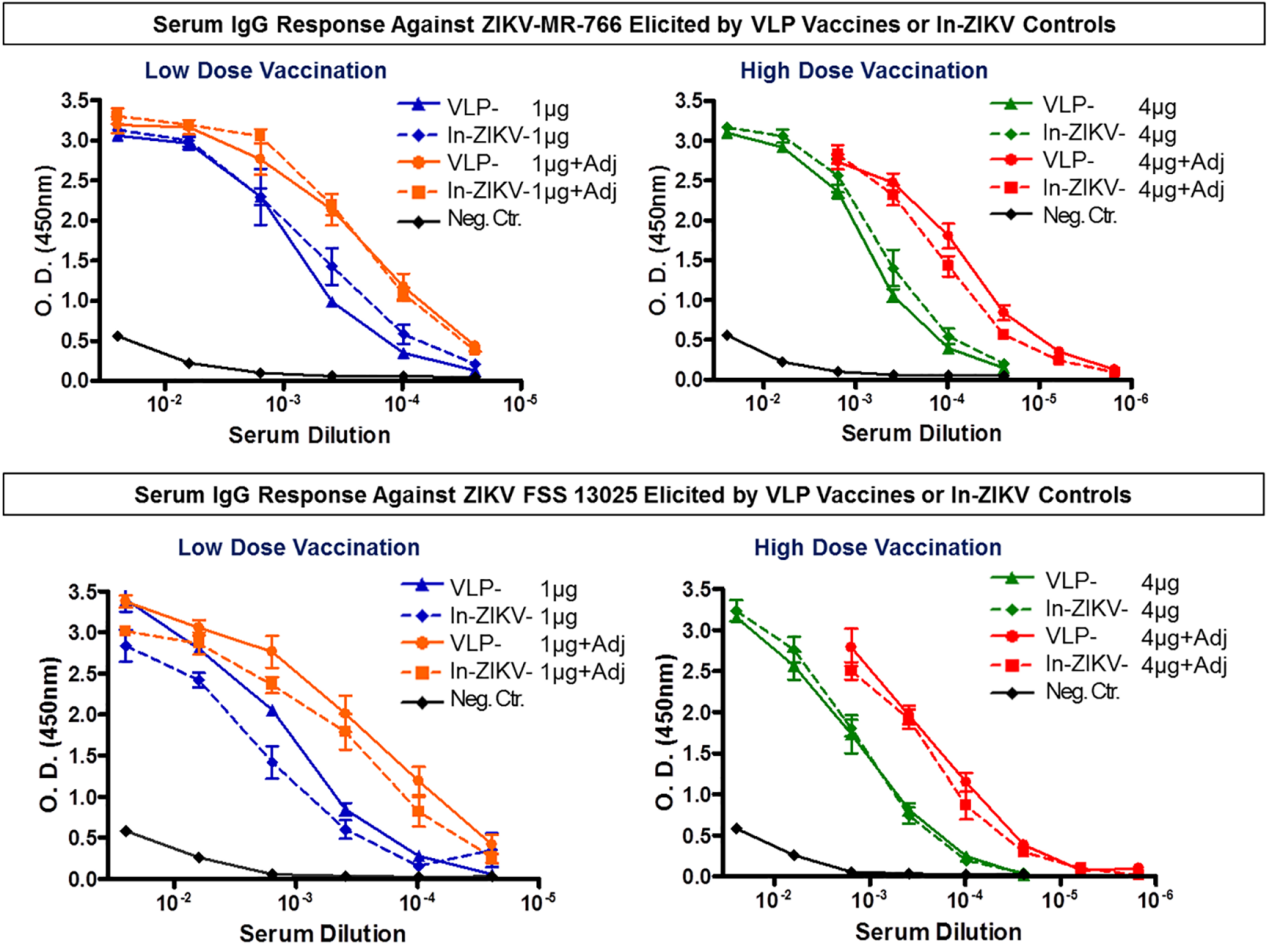
Evaluation of serum IgG response elicited by VLP and control vaccinations. Serum antibody (total IgG) elicited by the Zika VLP vaccine (VLP), inactivated Zika virus (In-ZIKV) and negative control (Neg. Ctr.) were measured by ELISA. Groups of BALB/c mice (n = 8) were immunized on days 0 and 21 with a VLP-based Zika vaccine (VLP), or an inactivated Zika virus vaccine (In-ZIKV) at doses of 1ug or 4ug with or without adjuvant. Blood samples were collected three weeks after the booster immunization and total serum IgG was measured via ELISA using as antigens two different Zika viruses MR766 (Upper panel) and FSS-13025 (Lower panel). Mice that received low dose (1μg alone or plus adjuvant) or high dose (4μg alone or plus adjuvant) of either vaccine showed a strong antibody response to the MR-766 virus compared to the placebo group (Upper panel). Mice showed high titers of IgG antibodies against FSS-13025 Zika virus as well (Lower panel). The values shown represent geometric means of the reciprocal dilutions of each mouse serum and data point standard deviations.

We assessed the level of the total serum IgG response in all vaccinated animals by ELISA using as antigens two Zika virus strains, one isolated in 1947 (Zika virus MR-766) and a second more current strain isolated in Cambodia in 2013 (Zika virus, FSS 13025). The ELISA results demonstrated that mice vaccinated with the low dose (1 μg) or high dose (4 μg) of VLP vaccine stimulated the production of high levels of serum antibodies against both Zika virus strains (Fig 6 Upper and Lower panels). This response was enhanced when the VLP vaccines were formulated with adjuvant. The 1 μg VLP vaccine plus adjuvant elicited a markedly increased level of serum IgG as compared to VLP alone. This enhancement was even more significant using the 4 μg VLP vaccine dose formulated with adjuvant. Similar results were obtained with the two Zika virus antigens used in the ELISA studies (Fig 6 Upper and Lower panels). Mice vaccinated with the inactivated Zika virus (In-ZIKV) produced a serum IgG response that was quite comparable to the one triggered by the VLP vaccines.

Clearly, both the VLP and the inactivated Zika virus control compositions were capable of eliciting a high antibody response against two Zika viruses. In contrast, the negative control group did not demonstrate a specific IgG response against Zika.

### Assessment of VLP vaccine neutralization efficacy

**Fig 7:**
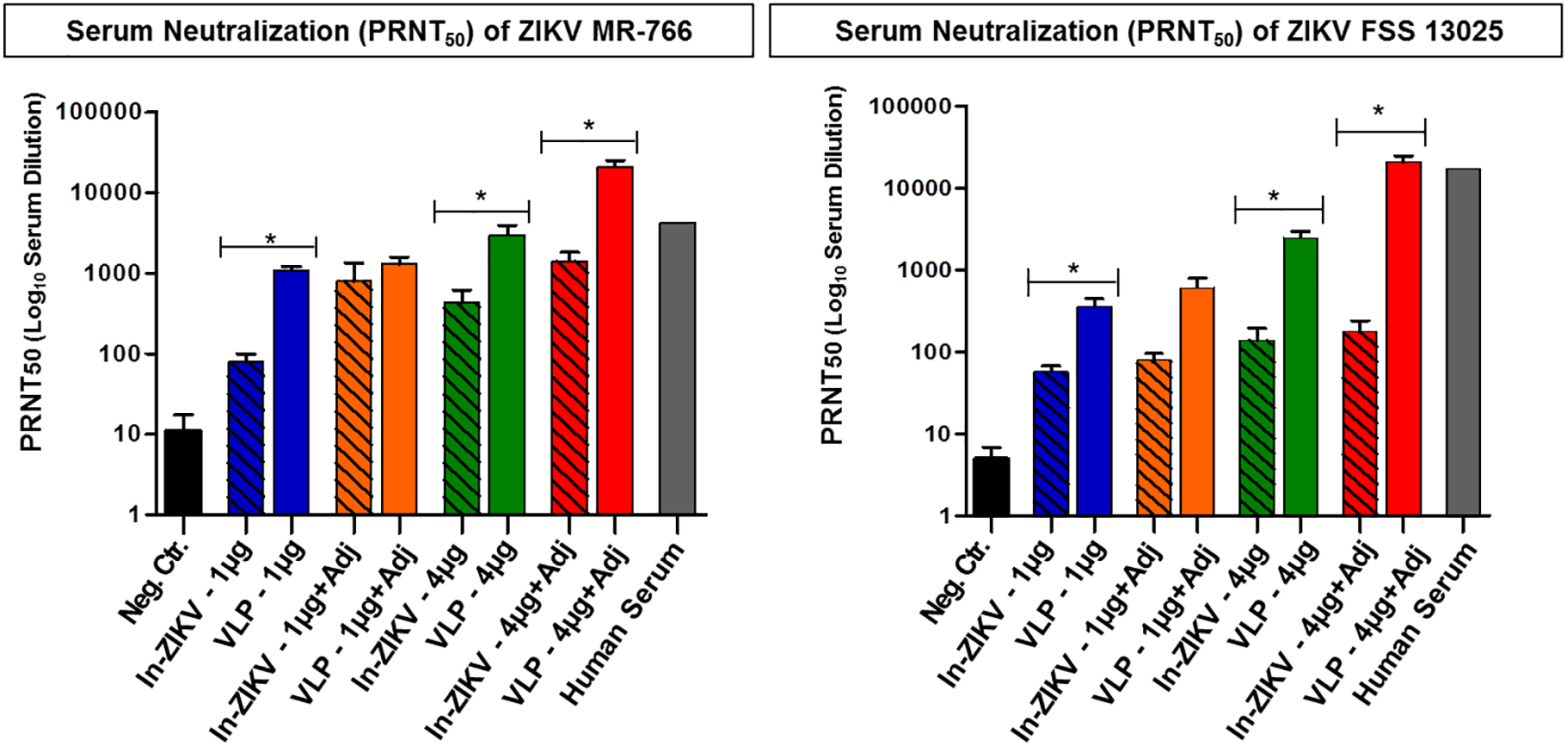
Serum neutralizing antibody responses by plaque reduction neutralization test (PRNT) against two Zika viruses. The serum neutralizing activity was plotted as PRNT50. (**Left Panel)** PRNT with MR-766 showed that the VLP vaccines elicited significantly higher neutralizing titers than equivalent composition of In-ZIKV vaccine. The VLP 1 μg plus adjuvant elicits higher titers than the corresponding In-ZIKV vaccine but the difference was not significant. The VLP (4μg+ adj) showed titers 5 fold greater than the human serum and the 4 μg alone was within this range, however statistical analysis was not feasible with only one human sample. (**Right Panel)** Similarly, PRNT with FSS-13025 showed that VLP vaccines exhibit stronger neutralization than the corresponding In-ZIKV vaccine. Neutralizing titers elicited by the In-ZIKV vaccine against FSS-13025 were much lower than those seen with the MR-766 and this may be due to the fact the In-ZIK vaccine was prepared with the MR-766 virus, which elicited a higher neutralizing titer toward the analogous virus (MR-766) than to a more distant one (FSS-13025). Similarly, the human serum showed a higher titer against FSS-13025 than MR-766, presumably because it resulted from a human infection in Brazil with a virus antigenically related to the FSS-13025. P-Values indicates t-tests.

Elicitation of high titers of specific neutralizing antibodies has been found to correlate with protection against several flaviviruses including yellow fever (YF) tick-borne encephalitis, dengue and Japanese encephalitis [35–37]. Although this has not yet been established for Zika it is very likely that the presence of high titers of neutralizing antibodies confer protection to infection. Therefore, we assessed the level of neutralizing antibodies elicited by the VLP vaccine and controls using a plaque reduction neutralization test (PRNT) with the two Zika viruses previously described. This evaluation with the ZIKV MR-766 showed that the VLP vaccine at the lower dose (1 μg) elicited neutralizing antibody titers significantly higher than the negative control group, which corresponds to a PRNT50: 1085 for the vaccine versus PRNT50: <25 for the negative control (Fig 7 Left panel and Table 1). This response was enhanced when adjuvant was part of the formulation raising the PRNT50: 1301. In contrast, the inactivated Zika virus (In-ZIKV) control at equivalent dose and formulation stimulated much lower neutralizing titers than the VLP vaccine reaching PRNT50: 79 for the 1 μg dose and PRNT50: 794 for the 1 μg plus adjuvant formulation. Increasing the VLP vaccine dose to 4 μg with or without adjuvant significantly raised the neutralizing antibody titers to PRNT50: 2978 and PRNT50: 20854, respectively. Including the adjuvant in the 4 μg VLP vaccine formulation heightened neutralization titers seven fold. The In-ZIKV high dose (4 μg) control showed improved titers when adjuvant was added PRNT50: 1408 versus PRNT50: 430 for In-ZIKV alone, but neither formulation gives rise to the neutralization activity attained with the VLP vaccine. All VLP vaccine formulations elicited statistically significant higher neutralizing antibodies titers than the equivalent compositions of the inactivated Zika virus vaccine with the exception of the 1 μg plus adjuvant dose, where the VLP induced higher titers but the difference was not significant. Neutralization analysis with the more contemporaneous ZIKV FSS-13025 resulted in a neutralization pattern that resembled the one seen with ZIKV MR-766. The VLP vaccine showed significantly higher neutralizing titers against ZIKV FSS-13025 than the In-ZIKV vaccine and this increased with the rise in dose and the incorporation of adjuvant (Fig 7 Right panel and Table 1). Furthermore, we used as a reference in the PRNT50 a human serum from patient who had recovered from a Zika infection. This revealed high titers of neutralizing antibody against the two Zika viruses tested; although the neutralizing activity was 4-fold higher for ZIKV-FSS virus (PRNT50: 17,067 for FSS versus and PRNT50: 4267 for MR-766) (Fig 7 Left and Right panels and Table 1). Since the human serum was obtained from a recently infected patient, it is likely that it better neutralized the more contemporaneous ZIKV–FSS suggesting that differences in neutralizing epitopes may exist between these two viruses. Given the fact that until now we have had access to only one Zika infected human serum sample, we could not perform a more comprehensive comparison with the VLP vaccine. Nonetheless, these results showed that the VLP vaccine was superior in eliciting neutralizing antibody responses than equivalent compositions of an In-ZIKV control (Fig 7 Left and Right panels and Table 1).

**Table 1:** Comparison of the neutralizing antibody responses (PRNT50) elicited by the VLP vaccine and controls. A summary of geometric mean titers of PRNT_50_ GMT for comparison between the two ZIKV strains, MR-766 and FSS 13025 for both vaccine and virus control groups. Neutralizing activity elicited by the VLP vaccines against the two viruses were comparable, except the VLP for 1μg alone, which had greater activity against MR766 than that of FSS (^**a**^ *p = 0*.*0033*). The In-ZIK virus control 4 μg plus adjuvant also showed a difference between the two viruses (^**b**^ *p = 0*.*026*). The statistical analysis was performed using t-test at confidence intervals of 95% with GraphPad Prism 4 software. Human serum isolated from a patient who recovered from a Zika infection was used to validate PRNT technique. However, no statistical analysis can be performed to compare the neutralization with two different Zika viruses due to the low sample size.

Neutralizing Antibody Titers in VLP Vaccinated and Control Groups				
Vaccine	Dose (μg)	Adjuvant	PRNT50 MR766	PRNT50 FSS13025
Neg. Ctr.	-	PBS + ADJ	11	5
VLP Vaccine	1	No ADJ	1,085 ^**a**^	357 ^**a**^
	1	ADJ	1,301	605
	4	No ADJ	2,978	2,476
	4	ADJ	20,854	20,826
In-ZIKV	1	No ADJ	79	57
	1	ADJ	794	79
	4	No ADJ	430	160
	4	ADJ	1,408 ^**b**^	177 ^**b**^
Human serum from Zika patient, Brazil 2013			4,267	17,067

**Fig 8:**
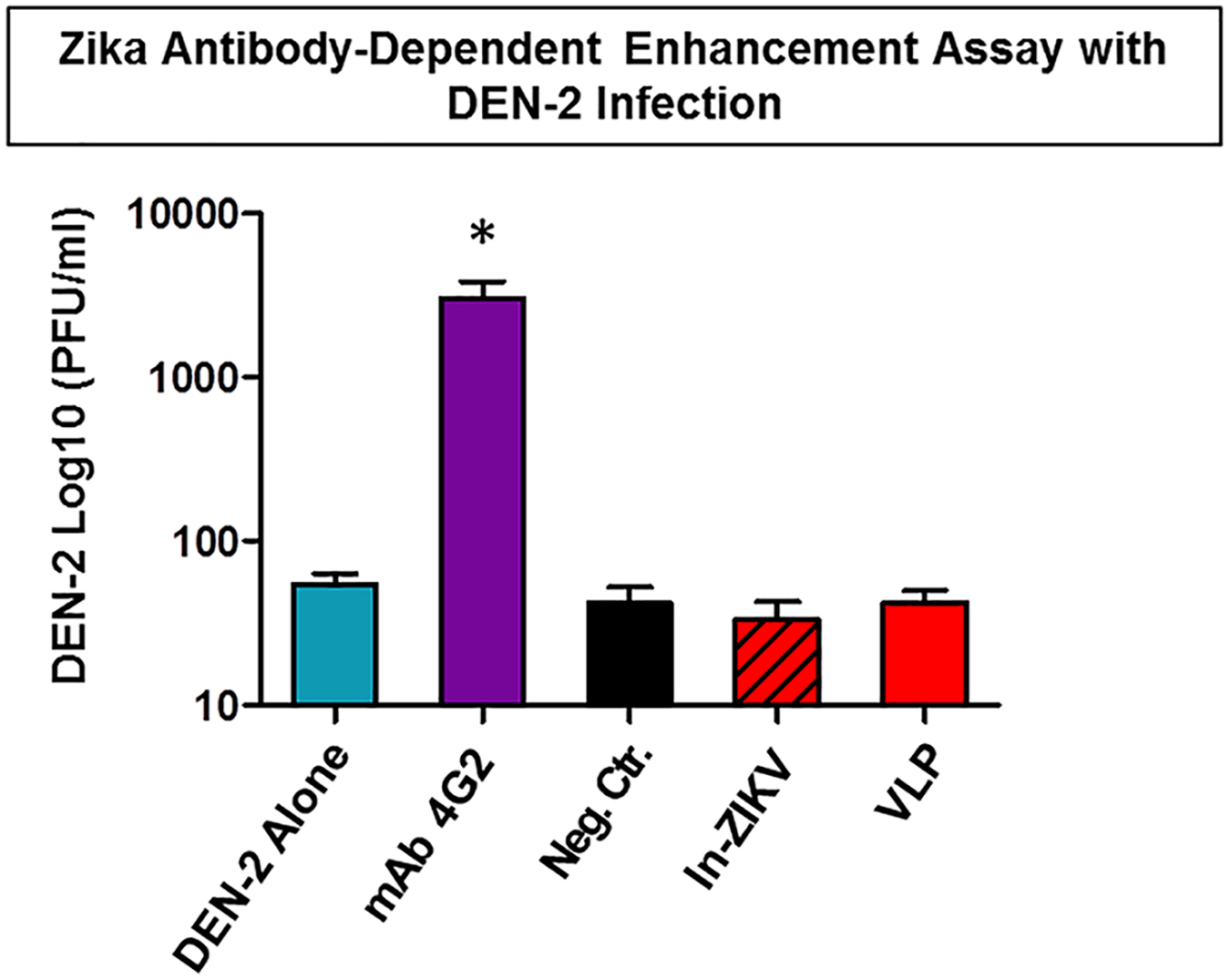
Antibody dependent enhancement (ADE) of dengue virus infection by the anti-ZIKV response elicited by vaccination. Serum from four VLP immunized mice (4ug +adj); inactivated ZIK (4ug+adj) and negative control (PBS+adj) diluted 1/500 were mixed with DENV-2 virus and added to U397 cells. The MAb 4G2 and virus alone were used as controls. After 96h incubation, DENV-2 virus titers were measured on the culture supernatant by plaque assay. Error bars indicate standard deviation of the four samples performed in duplicates. Asterisk (*) indicates that difference between 4G2 (positive control) and remaining samples is statistically significant (*p* < 0.05).

Considering that flavivirus infections may induce antibody-dependent enhancement of infection (ADE) with heterologous/co-circulating viruses, we tested whether the antibody response elicited by the VLP or controls vaccination against ZIKV may play a role in augmenting dengue virus infection. Here, we utilized an in vitro ADE assay [25] and measured whether DENV-2 infection was enhanced in the presence of anti-ZIKV antibodies. We used the MAb 4G2, which enhances dengue virus infections as positive control and DENV-2 alone as reference. This study showed that the antibody response to ZIKV elicited by VLP vaccination did not enhance DENV-2 infection (Fig 8). Nor did the sera from the In-ZIKV and the negative control vaccination when compared to DENV-2 with 4G2 MAb, which serves as the positive control and significantly enhanced DENV-2 infection (Fig 8). These data suggest that the antibody response elicited by these ZIKV vaccines at the dilution tested in the assay did not induce ADE of dengue 2-virus infection.

## Discussion

The ongoing Zika epidemic has already resulted in over 1500 cases of microcephaly in Brazil and 15 cases in the continental United States born to mothers who had traveled to affected areas [38, 39]. Many other countries in South America and the Caribbean are also experiencing the effects of the Zika epidemics and some autochthonous infections have been reported in the continental US in Florida. In addition to the mosquito vector direct infection there appear to be other forms of transmission including via sexual contact [11, 13] potentially increasing the risk of viral dissemination. Developing a safe and effective vaccine to control and combat the spread of the Zika virus is a high priority. Our work describes a new strategy to generate Zika virus-like particles (VLPs), providing a potentially safe and effective platform for the rapid production of a candidate for clinical development of a prophylactic Zika vaccine. The single co-expression of the structural polyprotein CprME together with the non-structural NS2B/NS3Pro suffices for the processing of CprME leading to the self-assembly and release of VLPs devoid of viral RNA. Purified VLPs resemble the wild type Zika virus in size, morphology and antigenic composition offering a suitable strategy for vaccine development. Several highly effective viral vaccines are based on the VLP strategy [40, 41] providing rationale for the use of Zika VLP for vaccine development. VLP vaccine production can be attained in suspension cultures of mammalian cells using standard fermentation technology. Considering that VLPs are not infectious or able to replicate, inactivation is not required better preserving protein structure and conformational epitopes. Our results show that all VLP vaccine and In-ZIKV control formulations elicited high titers of serum IgG antibodies. However these high levels of Zika specific antibody did not correlate, in the In-ZIKV vaccine control, with induction of high titers of neutralizing antibodies, which are the benchmark of protection. Most of our VLP vaccine formulations stimulated neutralizing antibody titers that were significantly higher than those induced by the In-ZIKV control. The discrepancy between high ELISA titers and low neutralization response in the In-ZIKV control, as compared to the VLP vaccine, may be attributed to the distortion of critical neutralizing epitopes as a result of ZIKV inactivation, which may reduce the repertoire of neutralizing sites. We have seen some of these effects where formalin inactivation of the Zika viruses abrogated most of the reactivity with the MAbs 4G2, ZV-48, ZV-64 and ZV-67, which recognize conformational epitopes in the fusion loop of domain II and domain III of the E protein (Fig 4). Therefore, the display of native and properly configured proteins on the surfaces of the VLPs appears to exhibit to the immune system a better antigenic array, which promotes the stimulation of a more diverse neutralizing response as we have seen with these VLP vaccine formulations.

Suboptimal neutralizing antibody levels may contribute to antibody-enhancement of disease in dengue, which by phylogenetic analysis is closer to Zika than other flaviviruses. In fact, one study has shown that combining dengue cross-reacting antibodies with Zika virus enhanced Zika infection in a cell culture assay [42]. In recent years, quaternary epitopes identified on the surface of dengue have been recognized as strong neutralizing sites [43–46]. Furthermore, a recent study has shown that monoclonal antibodies recognizing these highly neutralizing epitopes of dengue have also shown protection against Zika highlighting the significance of these types of epitopes in providing protection against Zika virus infection [47]. Although the correlates of protection for a Zika vaccine remain to be established, the elicitation of a strong and diverse neutralizing response may greatly contribute to protection against Zika infection. As shown in this study, the VLP based Zika vaccine elicited a robust neutralizing antibody response suggesting that critical conformational epitopes were indeed displayed for effective immune stimulation. In contrast to the response elicited by our VLPs, the In-ZIKV produced a much-reduced neutralizing response, even though the ELISA titers were as high as those induced by the VLP vaccine, suggesting that important neutralizing epitopes were not properly displayed in the In-ZIKV composition.

To investigate the possibility that the anti-ZIKV antibodies elicited by VLP vaccination may partially bind to dengue virus and contribute to antibody-dependent enhancement (ADE) of dengue infection, we tested diluted serum samples of VLP and control immunized animals in an ADE in vitro assay. This evaluation demonstrated lack of enhancement of DENV-2 infection when either the VLP vaccine or control immunizations stimulated antibodies were tested. In contrast, the 4G2 MAb significantly enhanced dengue infection, a property well established for this antibody [42]. This outcome provides some indication that ZIKV VLP vaccination does not appear to mediate ADE at least with DENV-2.

This study describes a safe, effective and straightforward strategy to rapidly produce a Zika vaccine. VLPs are produced in mammalian suspension cell cultures offering a suitable system for rapid scale up of manufacturing, without the risk of working with an infectious agent. The lack of infectivity of the product eliminates the need for chemical inactivation, which may compromise vaccine efficacy and safety. The particulate nature of the vaccine and the preservation of a variety of conformational antigenic sites may even render this vaccine efficacious in humans without using adjuvant if its incorporation into a vaccine raises safety concerns. In summary, the Zika VLP platform puts forward a vaccine composition and production system ready for clinical development of a safe and effective prophylactic Zika vaccine, which is greatly needed to meet the challenges imposed by the spread of the Zika epidemic.

## References

[1] Dick GW, Kitchen SF, Haddow AJ. Zika virus. I. Isolations and serological specificity. Trans R Soc Trop Med Hyg. 1952;46(5):509–20. 12995440 10.1016/0035-9203(52)90042-4

[2] Duffy MR, Chen TH, Hancock WT, Powers AM, Kool JL, Lanciotti RS, et al Zika virus outbreak on Yap Island, Federated States of Micronesia. The New England journal of medicine. 2009;360(24):2536–43. doi: 10.1056/NEJMoa0805715 19516034 10.1056/NEJMoa0805715

[3] Lanciotti RS, Kosoy OL, Laven JJ, Velez JO, Lambert AJ, Johnson AJ, et al Genetic and serologic properties of Zika virus associated with an epidemic, Yap State, Micronesia, 2007. Emerging infectious diseases. 2008;14(8):1232–9. PubMed Central PMCID: PMCPMC2600394. doi: 10.3201/eid1408.080287 18680646 10.3201/eid1408.080287 PMC2600394

[4] Haddow AD, Schuh AJ, Yasuda CY, Kasper MR, Heang V, Huy R, et al Genetic characterization of Zika virus strains: geographic expansion of the Asian lineage. PLoS neglected tropical diseases. 2012;6(2):e1477 PubMed Central PMCID: PMCPMC3289602. doi: 10.1371/journal.pntd.0001477 22389730 10.1371/journal.pntd.0001477 PMC3289602

[5] Faye O, Freire CC, Iamarino A, Faye O, de Oliveira JV, Diallo M, et al Molecular evolution of Zika virus during its emergence in the 20(th) century. PLoS neglected tropical diseases. 2014;8(1):e2636 PubMed Central PMCID: PMCPMC3888466. doi: 10.1371/journal.pntd.0002636 24421913 10.1371/journal.pntd.0002636 PMC3888466

[6] Zanluca C, de Melo VC, Mosimann AL, Dos Santos GI, Dos Santos CN, Luz K. First report of autochthonous transmission of Zika virus in Brazil. Mem Inst Oswaldo Cruz. 2015;110(4):569–72. PubMed Central PMCID: PMCPMC4501423. doi: 10.1590/0074-02760150192 26061233 10.1590/0074-02760150192 PMC4501423

[7] CDC. Zika virus disease in the United States, 2015–2016. 2016.

[8] Valentine G, Marquez L, Pammi M. Zika Virus-Associated Microcephaly and Eye Lesions in the Newborn. J Pediatric Infect Dis Soc. 2016. 10.1093/jpids/piw037 27405738

[9] Cao-Lormeau VM, Blake A, Mons S, Lastere S, Roche C, Vanhomwegen J, et al Guillain-Barre Syndrome outbreak associated with Zika virus infection in French Polynesia: a case-control study. Lancet. 2016;387(10027):1531–9. doi: 10.1016/S0140-6736(16)00562-6 26948433 10.1016/S0140-6736(16)00562-6 PMC5444521

[10] Foy BD, Kobylinski KC, Chilson Foy JL, Blitvich BJ, Travassos da Rosa A, Haddow AD, et al Probable non-vector-borne transmission of Zika virus, Colorado, USA. Emerging infectious diseases. 2011;17(5):880–2. PubMed Central PMCID: PMCPMC3321795. doi: 10.3201/eid1705.101939 21529401 10.3201/eid1705.101939 PMC3321795

[11] D Ortenzio E, Matheron S, Yazdanpanah Y, de Lamballerie X, Hubert B, Piorkowski G, et al Evidence of Sexual Transmission of Zika Virus. The New England journal of medicine. 2016;374(22):2195–8. doi: 10.1056/NEJMc1604449 27074370 10.1056/NEJMc1604449

[12] Musso D, Roche C, Nhan TX, Robin E, Teissier A, Cao-Lormeau VM. Detection of Zika virus in saliva. J Clin Virol. 2015;68:53–5. doi: 10.1016/j.jcv.2015.04.021 26071336 10.1016/j.jcv.2015.04.021

[13] Fourcade C, Mansuy JM, Dutertre M, Delpech M, Marchou B, Delobel P, et al Viral load kinetics of Zika virus in plasma, urine and saliva in a couple returning from Martinique, French West Indies. J Clin Virol. 2016;82:1–4. doi: 10.1016/j.jcv.2016.06.011 27389909 10.1016/j.jcv.2016.06.011

[14] Gourinat AC, O'Connor O, Calvez E, Goarant C, Dupont-Rouzeyrol M. Detection of Zika virus in urine. Emerging infectious diseases. 2015;21(1):84–6. PubMed Central PMCID: PMCPMC4285245. doi: 10.3201/eid2101.140894 25530324 10.3201/eid2101.140894 PMC4285245

[15] Lindenbach BD, Murray C.L., Thiel H-J., Rice C.M. Flaviviridae. Fields Virology, Sixth Edition, Lippincott Williams & Wilkins 2013;Vol. I, Chapter 25:713–46.

[16] Baronti C, Piorkowski G, Charrel RN, Boubis L, Leparc-Goffart I, de Lamballerie X. Complete coding sequence of zika virus from a French polynesia outbreak in 2013. Genome Announc. 2014;2(3). PubMed Central PMCID: PMCPMC4047448. 10.1128/genomeA.00500-14 PMC4047448 24903869

[17] Crill WD, Chang GJ. Localization and characterization of flavivirus envelope glycoprotein cross-reactive epitopes. Journal of virology. 2004;78(24):13975–86. PubMed Central PMCID: PMCPMC533943. doi: 10.1128/JVI.78.24.13975-13986.2004 15564505 10.1128/JVI.78.24.13975-13986.2004 PMC533943

[18] Larocca RA, Abbink P, Peron JP, Zanotto PM, Iampietro MJ, Badamchi-Zadeh A, et al Vaccine protection against Zika virus from Brazil. Nature. 2016. 10.1038/nature18952 PMC5003703 27355570

[19] Eckels KH, Putnak R. Formalin-inactivated whole virus and recombinant subunit flavivirus vaccines. Advances in virus research. 2003;61:395–418. 14714438 10.1016/s0065-3527(03)61010-9

[20] Zhao H, Fernandez E, Dowd KA, Speer SD, Platt DJ, Gorman MJ, et al Structural Basis of Zika Virus-Specific Antibody Protection. Cell. 2016;166(4):1016–27. PubMed Central PMCID: PMCPMC4983199. doi: 10.1016/j.cell.2016.07.020 27475895 10.1016/j.cell.2016.07.020 PMC4983199

[21] Gentry MK, Henchal EA, McCown JM, Brandt WE, Dalrymple JM. Identification of distinct antigenic determinants on dengue-2 virus using monoclonal antibodies. The American journal of tropical medicine and hygiene. 1982;31(3 Pt 1):548–55. 6177259 10.4269/ajtmh.1982.31.548

[22] Timiryasova TM, Bonaparte MI, Luo P, Zedar R, Hu BT, Hildreth SW. Optimization and validation of a plaque reduction neutralization test for the detection of neutralizing antibodies to four serotypes of dengue virus used in support of dengue vaccine development. The American journal of tropical medicine and hygiene. 2013;88(5):962–70. PubMed Central PMCID: PMCPMC3752766. doi: 10.4269/ajtmh.12-0461 23458954 10.4269/ajtmh.12-0461 PMC3752766

[23] WHO. Guidelines for Plaque-Reduction Neutralization Testing of human antibodies to Dengue viruses. 2007. 10.1089/vim.2008.0007 18476771

[24] Finney DJ. Probit Analysis. Ed,Cambridge, England, Cambridge University Press 1952.

[25] Diamond MS, Edgil D, Roberts TG, Lu B, Harris E. Infection of human cells by dengue virus is modulated by different cell types and viral strains. Journal of virology. 2000;74(17):7814–23. PubMed Central PMCID: PMCPMC112311. 10933688 10.1128/jvi.74.17.7814-7823.2000 PMC112311

[26] Smith TJ, Brandt WE, Swanson JL, McCown JM, Buescher EL. Physical and biological properties of dengue-2 virus and associated antigens. Journal of virology. 1970;5(4):524–32. PubMed Central PMCID: PMCPMC376035. 4195055 10.1128/jvi.5.4.524-532.1970 PMC376035

[27] Schalich J, Allison SL, Stiasny K, Mandl CW, Kunz C, Heinz FX. Recombinant subviral particles from tick-borne encephalitis virus are fusogenic and provide a model system for studying flavivirus envelope glycoprotein functions. Journal of virology. 1996;70(7):4549–57. PubMed Central PMCID: PMCPMC190391. 8676481 10.1128/jvi.70.7.4549-4557.1996 PMC190391

[28] Allison SL, Stadler K, Mandl CW, Kunz C, Heinz FX. Synthesis and secretion of recombinant tick-borne encephalitis virus protein E in soluble and particulate form. Journal of virology. 1995;69(9):5816–20. PubMed Central PMCID: PMCPMC189449. 7637027 10.1128/jvi.69.9.5816-5820.1995 PMC189449

[29] Allison SL, Tao YJ, O'Riordain G, Mandl CW, Harrison SC, Heinz FX. Two distinct size classes of immature and mature subviral particles from tick-borne encephalitis virus. Journal of virology. 2003;77(21):11357–66. PubMed Central PMCID: PMC229348. doi: 10.1128/JVI.77.21.11357-11366.2003 14557621 10.1128/JVI.77.21.11357-11366.2003 PMC229348

[30] Suphatrakul A, Yasanga T, Keelapang P, Sriburi R, Roytrakul T, Pulmanausahakul R, et al Generation and preclinical immunogenicity study of dengue type 2 virus-like particles derived from stably transfected mosquito cells. Vaccine. 2015;33(42):5613–22. doi: 10.1016/j.vaccine.2015.08.090 26382602 10.1016/j.vaccine.2015.08.090

[31] Sirohi D, Chen Z, Sun L, Klose T, Pierson TC, Rossmann MG, et al The 3.8 A resolution cryo-EM structure of Zika virus. Science. 2016;352(6284):467–70. PubMed Central PMCID: PMCPMC4845755. doi: 10.1126/science.aaf5316 27033547 10.1126/science.aaf5316 PMC4845755

[32] Kostyuchenko VA, Lim EX, Zhang S, Fibriansah G, Ng TS, Ooi JS, et al Structure of the thermally stable Zika virus. Nature. 2016;533(7603):425–8. 27093288 10.1038/nature17994

[33] Khurana S, Verma N, Yewdell JW, Hilbert AK, Castellino F, Lattanzi M, et al MF59 adjuvant enhances diversity and affinity of antibody-mediated immune response to pandemic influenza vaccines. Sci Transl Med. 2011;3(85):85ra48 PubMed Central PMCID: PMCPMC3501657. doi: 10.1126/scitranslmed.3002336 21632986 10.1126/scitranslmed.3002336 PMC3501657

[34] O Hagan DT, Ott GS, Nest GV, Rappuoli R, Giudice GD. The history of MF59((R)) adjuvant: a phoenix that arose from the ashes. Expert review of vaccines. 2013;12(1):13–30. doi: 10.1586/erv.12.140 23256736 10.1586/erv.12.140

[35] Belmusto-Worn VE, Sanchez JL, McCarthy K, Nichols R, Bautista CT, Magill AJ, et al Randomized, double-blind, phase III, pivotal field trial of the comparative immunogenicity, safety, and tolerability of two yellow fever 17D vaccines (Arilvax and YF-VAX) in healthy infants and children in Peru. The American journal of tropical medicine and hygiene. 2005;72(2):189–97. 15741556

[36] Heinz FX, Holzmann H, Essl A, Kundi M. Field effectiveness of vaccination against tick-borne encephalitis. Vaccine. 2007;25(43):7559–67. doi: 10.1016/j.vaccine.2007.08.024 17869389 10.1016/j.vaccine.2007.08.024

[37] Katzelnick LC, Montoya M, Gresh L, Balmaseda A, Harris E. Neutralizing antibody titers against dengue virus correlate with protection from symptomatic infection in a longitudinal cohort. Proceedings of the National Academy of Sciences of the United States of America. 2016;113(3):728–33. PubMed Central PMCID: PMCPMC4725482. doi: 10.1073/pnas.1522136113 26729879 10.1073/pnas.1522136113 PMC4725482

[38] CDC. Outcomes of Pregnancies with Laboratory Evidence of Possible Zika Virus Infection in the United States, 2016. 2016.

[39] PAHO. Regional Zika Epidemiology Update (Americas) July, 29, 2016. 2016.

[40] Joura EA, Leodolter S, Hernandez-Avila M, Wheeler CM, Perez G, Koutsky LA, et al Efficacy of a quadrivalent prophylactic human papillomavirus (types 6, 11, 16, and 18) L1 virus-like-particle vaccine against high-grade vulval and vaginal lesions: a combined analysis of three randomised clinical trials. Lancet. 2007;369(9574):1693–702. doi: 10.1016/S0140-6736(07)60777-6 17512854 10.1016/S0140-6736(07)60777-6

[41] Zhang J, Zhang XF, Huang SJ, Wu T, Hu YM, Wang ZZ, et al Long-term efficacy of a hepatitis E vaccine. The New England journal of medicine. 2015;372(10):914–22. doi: 10.1056/NEJMoa1406011 25738667 10.1056/NEJMoa1406011

[42] Dejnirattisai W, Supasa P, Wongwiwat W, Rouvinski A, Barba-Spaeth G, Duangchinda T, et al Dengue virus sero-cross-reactivity drives antibody-dependent enhancement of infection with zika virus. Nature immunology. 2016. 10.1038/ni.3515 PMC4994874 27339099

[43] de Alwis R, Smith SA, Olivarez NP, Messer WB, Huynh JP, Wahala WM, et al Identification of human neutralizing antibodies that bind to complex epitopes on dengue virions. Proceedings of the National Academy of Sciences of the United States of America. 2012;109(19):7439–44. PubMed Central PMCID: PMCPMC3358852. doi: 10.1073/pnas.1200566109 22499787 10.1073/pnas.1200566109 PMC3358852

[44] Rouvinski A, Guardado-Calvo P, Barba-Spaeth G, Duquerroy S, Vaney MC, Kikuti CM, et al Recognition determinants of broadly neutralizing human antibodies against dengue viruses. Nature. 2015;520(7545):109–13. doi: 10.1038/nature14130 25581790 10.1038/nature14130

[45] Fibriansah G, Tan JL, Smith SA, de Alwis R, Ng TS, Kostyuchenko VA, et al A highly potent human antibody neutralizes dengue virus serotype 3 by binding across three surface proteins. Nat Commun. 2015;6:6341 PubMed Central PMCID: PMCPMC4346626. doi: 10.1038/ncomms7341 25698059 10.1038/ncomms7341 PMC4346626

[46] Gallichotte EN, Widman DG, Yount BL, Wahala WM, Durbin A, Whitehead S, et al A new quaternary structure epitope on dengue virus serotype 2 is the target of durable type-specific neutralizing antibodies. MBio. 2015;6(5):e01461–15. PubMed Central PMCID: PMCPMC4620467. doi: 10.1128/mBio.01461-15 26463165 10.1128/mBio.01461-15 PMC4620467

[47] Swanstrom JA, Plante JA, Plante KS, Young EF, McGowan E, Gallichotte EN, et al Dengue Virus Envelope Dimer Epitope Monoclonal Antibodies Isolated from Dengue Patients Are Protective against Zika Virus. MBio. 2016;7(4). PubMed Central PMCID: PMCPMC4958264. 10.1128/mBio.01123-16 PMC4958264 27435464

